# Link between blood–brain barrier disruption and microglial activation

**DOI:** 10.4103/NRR.NRR-D-25-00103

**Published:** 2025-07-05

**Authors:** Arjun Sapkota, Sebok K. Halder, Richard Milner

**Affiliations:** San Diego Biomedical Research Institute, San Diego, CA, USA

Cells of the central nervous system (CNS) are privileged in lying behind the blood–brain barrier (BBB). Unlike blood vessels in other organs, CNS blood vessels are unique in displaying high electrical resistance and low permeability. With this unique structure and function, the BBB prevents potentially harmful blood components such as serum proteins, inflammatory cytokines, and inflammatory leukocytes from entering the hallowed space of the CNS and wreaking havoc. In addition to these “tightness” properties, the BBB has an array of specialized transporters designed to import essential nutrients, such as amino acids and glucose into the CNS. It also has transporters that remove unwanted chemicals or drugs from the CNS called multidrug resistance proteins, such as P-glycoprotein. At the structural level, the BBB consists of a lining of endothelial cells firmly attached to a basement membrane (BM) containing high levels of the extracellular matrix (ECM) proteins collagen IV, laminin, fibronectin, and perlecan. Pericytes and astrocyte foot processes also play an important role in inducing and maintaining BBB properties. At the molecular level, the BBB relies on adhesion molecules (primarily integrins) that bind endothelial cells to the BM as well as adherens and tight junction protein complexes that form between adjacent endothelial cells (Zlokovic, 2008). The importance of the BBB is illustrated by the fact that its disruption is instrumental in the initiation and/or maintenance in almost all neurological diseases, including ischemic stroke, multiple sclerosis, vascular dementia, and Alzheimer’s disease. Aside from deteriorating during disease conditions, accumulating evidence suggests that BBB integrity also declines as a function of age (Senatorov et al., 2019).

Several years ago, we began studying how cerebral blood vessels respond to hypoxic insults. While it had previously been well documented that chronic mild hypoxia (8% O_2_) induces a strong angiogenic response in the CNS, we found this comes at a cost; transient BBB disruption. We also discovered that this disruption is associated with a marked microglial response in which microglia migrate, proliferate, activate, and aggregate around damaged blood vessels (Halder and Milner, 2019). Of high translational relevance, hypoxia-induced BBB disruption and associated microglial activation responses are greatly amplified in aged mice, as seen by a 5–10-fold increase in the density of leaky blood vessels and greatly enhanced microglial activation (Halder and Milner, 2022). When considering the clinical significance of these observations, it is aged that is particularly important because this is the group that experiences more frequent and severe levels of hypoxia due to the common age-related conditions of the heart (ischemic heart disease and heart failure) and lungs (pulmonary fibrosis, emphysema, asthma, and sleep apnea). In contrast to our demonstration of a close association between hypoxia-induced BBB disruption and microglial activation in mice, recent magnetic resonance imaging studies examining this relationship in patients with cerebral small vessel disease, found that BBB breakdown and microglial activation occur in a spatially independent manner (Walsh et al., 2021). Motivated by this apparent contradiction, we set out to examine the relationship between BBB disruption and microglial activation further, to ask some specific questions. First, how close is the relationship between BBB breakdown and microglial activation? Second, do hypoxia-disrupted cerebral blood vessels spontaneously repair or do they remain leaky permanently? Third, if damaged blood vessels spontaneously repair themselves, do the activated microglia return to their pre-hypoxic resting state?

**Figure 1:**
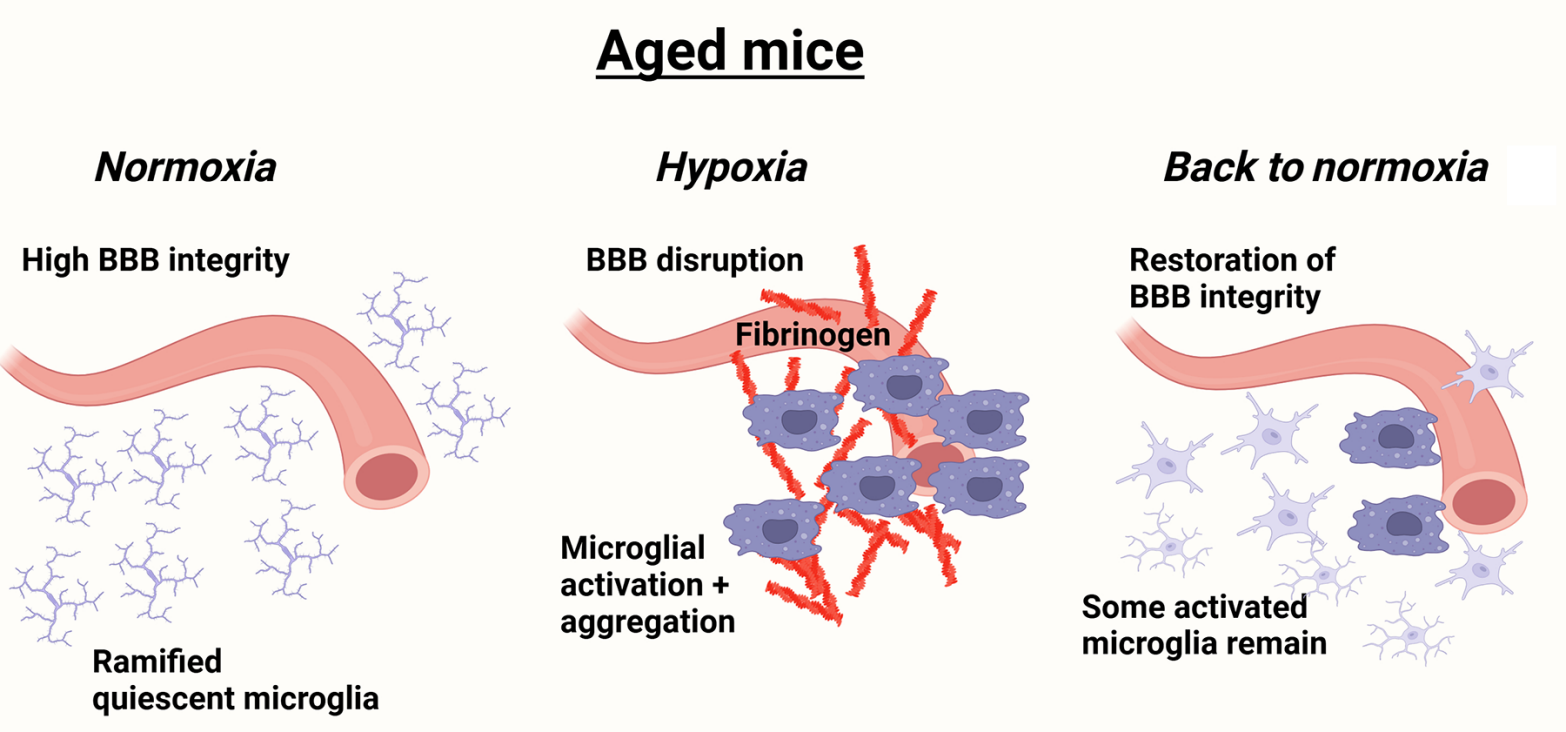
Under control normoxia conditions, cerebral blood vessels display strong blood–brain barrier (BBB) integrity and microglia occupy a predominantly resting ramified phenotype. Chronic mild hypoxia leads to BBB disruption and extravascular leak of fibrinogen, which promotes microglial activation. While subsequent return to normoxia conditions promotes repair of disrupted blood vessels and restoration of BBB integrity, microglia in the surrounding area are slow to return to their pre-hypoxic phenotype and remain chronically activated for some time. Created with BioRender.com.

To directly address these questions, in our recent study, we exposed aged (20 months old) mice to chronic mild hypoxia for 7 days, before analyzing the degree of BBB breakdown (assessed by extravascular deposition of fibrinogen and red blood cells) and microglial activation (Mac-1 and CD68), either immediately after hypoxic treatment or 7 or 14 days following the return of mice to normoxic conditions (Sapkota et al., 2025). The rationale behind this protocol was to try to model an aged patient who suffers an extended period of hypoxia due to cardiac or pulmonary disease but then attends the emergency room to receive oxygen and appropriate medication to restore oxygen saturation levels back to normal. Our first observation was that hypoxia-induced BBB disruption was always closely associated with a strong microglial response in which microglia activate and aggregate around disrupted blood vessels. Our second observation was that when mice were switched from hypoxia back to normoxic conditions, over the next 14 days, the extent of extravascular leak was greatly reduced, as shown by the total absence of extravascular leak of red blood cells (RBC) using the RBC marker TER-119 and greatly reduced levels of extravascular fibrinogen deposition. This demonstrates that return to normoxic conditions promotes spontaneous repair of the disrupted cerebral blood vessels. Our third observation was that despite the vascular repair, 14 days after mice were returned to normoxic conditions, microglia displayed a relatively activated phenotype as shown by morphological status and expression levels of Mac-1 and CD68 (**Figure 1**). In contrast, astrocyte activation, as assessed by glial fibrillary acidic protein signal intensity, had largely reverted to pre-hypoxic levels. Our fourth and final observation was that hypoxia-triggered BBB disruption was associated with marked loss of neurons in several areas of the brain, including the midbrain, cerebral cortex, and olfactory bulb. Of strong translational significance, these events were associated with a permanent decline in cognitive ability as assessed by the novel object recognition test.

All our mice studies performed to date suggest a strong relationship between disruption of the BBB and activation of surrounding microglia. This is supported not only by our data obtained in the relatively short-term chronic mild hypoxia model (14 days), but it is also evident when comparing young and aged mice, because greater BBB leak in aged mice correlates closely with greatly enhanced microglial activation, even under resting normoxic conditions. So why did not the recent magnetic resonance imaging study on patients with small vessel disease confirm these findings? Based on our recent findings of a temporal disconnect between BBB leakage and microglial activation upon return to normoxic conditions, we propose an alternative explanation that might account for the recent findings in patients. According to this model, at any moment in time, a newly erupting vascular leak may not have had time to sufficiently stimulate microglial activation, and conversely, a previous vascular leak that has largely repaired might still be surrounded by microglia still retaining signs of activation.

Taken together, our emerging findings suggest a model linking hypoxic exposure, BBB disruption, neuronal death, and cognitive decline. Our data has strong translational significance for aged patients because the chances of hypoxia are relatively high because of a myriad of medical conditions, including asthma, sleep apnea, chronic obstructive pulmonary disease, ischemic heart disease, and heart failure. Interestingly, prior studies have shown that many of these conditions increase the risk of cognitive decline (Yohannes et al., 2017), further supporting our concept that repeated hypoxic episodes may be an important pathogenic factor in the progression of cognitive decline and vascular dementia. Our recent findings also suggest another factor that exacerbates this hypoxia-dementia link, namely that even after hypoxia-damaged cerebral blood vessels have been repaired, surrounding microglia remain chronically activated for some time. This will serve to amplify neuroinflammation in the aged brain via the release of pro-inflammatory cytokines and chemokines, and the action of microglial proteases such as matrix metalloproteinase-9, which degrade ECM constituents of the vascular BM, further weakening the BBB.

It is important to acknowledge that the relationship between BBB disruption and microglial activation is not unique to insults of a hypoxic nature. Traumatic brain injury accounts for a vast number of injury-related deaths and its pathogenesis involves a secondary injury triggered by BBB disruption, leakage of blood components into the sensitive CNS parenchyma, and subsequent microglial activation. A recent study demonstrated a tight correlation between traumatic brain injury-induced BBB disruption (determined by IgG leak) and microglial activation (morphology of Iba-1^+^ cells) 1 and 7 days post-injury, but after 25 days post-injury, both IgG leak and microglial activation had declined (Green et al., 2024). Thus, it appears that in both hypoxia and traumatic brain injury, timely attenuation of BBB disruption and microglial activation may prove beneficial in preventing secondary neuroinflammatory events.

Because we have shown that microglia play an important vasculo-protective role following hypoxia-induced BBB disruption, it is important to recognize that the interaction between BBB stability and microglial activation is likely a two-way street. Hypoxia-induced BBB disruption in the aged presents a particular conundrum because our studies have shown that microglia are far more activated in aged mice compared to young, yet paradoxically, this correlates with greater levels of BBB breakdown. What might explain this paradox? Some insight was derived from our observation that in aged brains, despite microglia being more activated, they are much slower at surrounding and aggregating around vascular leaks. This suggested to us that a biphasic relationship may exist between microglial activation state and vasculo-protective function. In this model, microglia become activated to migrate and surround leaky blood vessels, but if microglia get overly activated (as in the aged brain), they lose their ability to migrate efficiently towards the leaking vessel and are slow to protect the vessel. To test this hypothesis, we treated aged mice with minocycline as a means of reducing microglial activation; this resulted in lower levels of microglial activation that correlated with reduced levels of BBB disruption. This demonstrates that manipulation (reduction) of microglial activation in the aged brain might present one way of promoting improved vasculo-protective function, thereby safeguarding the BBB. Systemic inflammation can also impact the BBB and microglia directly but also influence the nature of BBB-microglial interactions. Systemic inflammatory disease is associated with elevated levels of pro-inflammatory cytokines and chemokines, which will both weaken BBB integrity and activate microglia. Simultaneously, in autoimmune diseases such as multiple sclerosis, peripheral immune cells such as lymphocytes and monocytes cross the BBB and enter the CNS parenchyma, where their presence further activates microglia, thereby amplifying the neuroinflammatory state.

What relevance do these findings have for patients? One implication is that aged patients are much more vulnerable to the effects of hypoxia-induced BBB disruption than young people because (i) their BBB is ostensibly weaker than that of a younger person, and (ii) aged patients are much more likely to experience hypoxia from several different medical conditions. In addition, vasculo-protective functions of microglia in the aged brain are less efficient than in the young, which further adds to the pathogenic chain of events leading from hypoxia-BBB disruption-neurodegeneration-cognitive decline. With this information in hand, how can we improve the clinical outcome of aged patients exposed to hypoxia? One general guiding principle should be to delay the inevitable age-related deterioration of BBB integrity. At the molecular and cellular levels, several candidates come to mind. First, the ECM receptors, integrins play essential roles both in promoting strong endothelial adhesion to the underlying vascular BM and in mediating the regulatory influence of the ECM proteins contained within the vascular BM. Several studies show that the β1 class of integrins is essential for mediating these functions (Osada et al., 2011), raising the possibility that enhancing endothelial adhesion by increasing β1 integrin expression might prove beneficial in improving BBB integrity. Furthermore, as cerebral endothelial cells express several different β1 integrins, including α1β1, α3β1, α5β1, and α6β1 (Wang and Milner, 2006), all of which have distinct ECM ligand specificity (e.g., α1β1 is a collagen receptor; α5β1 binds fibronectin) and regulatory functions, a more beneficial approach to promoting BBB integrity might involve targeting of specific β1 integrins. Second, as endothelial tight junction proteins (such as ZO-1, claudin-5, and occludin) and adherens proteins (VE-cadherin) play critical roles in sealing inter-endothelial junctions at the BBB, enhancing expression of one or several of these components may also offer therapeutic benefit. Third, as accumulating evidence supports the idea that pericytes play a critical function in promoting BBB maturation and integrity along with recent findings that pericyte coverage at the BBB declines with age (Ding et al., 2021), approaches that promote pericyte coverage of cerebral blood vessels could also improve BBB integrity. Lastly, building on our finding that the highly activated microglia in aged mice are less effective at protecting blood vessels, as shown by reduced ability to migrate and aggregate around leaking blood vessels, but that pharmacological attenuation of microglial activation enhances their protective function, thereby reducing hypoxia-associated BBB breakdown (Halder and Milner, 2022), this suggests that a carefully titrated attenuation of microglial activation in aged patients might also prove effective in enhancing BBB integrity. How then might a better understanding of BBB integrity molecular mechanisms translate into practical clinical strategies? The first approach would be to identify drugs that are capable of increasing the expression or activation of specific endothelial integrins to enhance endothelial barrier function. A similar approach could be aimed at the tight junction and adherens proteins. As pericyte number dwindles with age, the use of specific growth factors that enhance pericyte proliferation and coverage such as platelet-derived growth factor BB also presents an attractive target. Finally, approaches that optimize microglial vasculo-protective function could include drugs that reduce microglial activation in the aged brain, such as minocycline as we have already demonstrated, but also more specific drugs such as low doses of PLX5622, which prevents microglia from becoming overly activated. Other microglia-targeted drugs could include inhibitors of specific metalloproteinases, such as matrix metalloproteinase-9 which has been shown to degrade BM ECM proteins and thus aid in BBB disruption. By thoroughly investigating all these potential therapeutic avenues, we are more likely to identify a strategy or combination of strategies that can delay the pathogenesis of hypoxia-induced BBB disruption and prevent the resulting vascular dementia.

*This work was supported by the NIH RF1 grant NS119477 jointly funded by NINDS and NIA (to RM).*
